# Infants’ Selectively Pay Attention to the Information They Receive from a Native Speaker of Their Language

**DOI:** 10.3389/fpsyg.2016.01150

**Published:** 2016-08-03

**Authors:** Hanna Marno, Bahia Guellai, Yamil Vidal, Julia Franzoi, Marina Nespor, Jacques Mehler

**Affiliations:** ^1^Department of Cognitive Science, Central European UniversityBudapest, Hungary; ^2^Language, Cognition and Development Lab, International School for Advanced StudiesTrieste, Italy; ^3^Laboratoire Ethologie Cognition, Université Paris Ouest Nanterre La DéfenseParis, France

**Keywords:** cultural knowledge, infants, native speakers, attention, social learning

## Abstract

From the first moments of their life, infants show a preference for their native language, as well as toward speakers with whom they share the same language. This preference appears to have broad consequences in various domains later on, supporting group affiliations and collaborative actions in children. Here, we propose that infants’ preference for native speakers of their language also serves a further purpose, specifically allowing them to efficiently acquire culture specific knowledge via social learning. By selectively attending to informants who are native speakers of their language and who probably also share the same cultural background with the infant, young learners can maximize the possibility to acquire cultural knowledge. To test whether infants would preferably attend the information they receive from a speaker of their native language, we familiarized 12-month-old infants with a native and a foreign speaker, and then presented them with movies where each of the speakers silently gazed toward unfamiliar objects. At test, infants’ looking behavior to the two objects alone was measured. Results revealed that infants preferred to look longer at the object presented by the native speaker. Strikingly, the effect was replicated also with 5-month-old infants, indicating an early development of such preference. These findings provide evidence that young infants pay more attention to the information presented by a person with whom they share the same language. This selectivity can serve as a basis for efficient social learning by influencing how infants’ allocate attention between potential sources of information in their environment.

## Introduction

Infants’ sensitivity to their native language has been shown shortly after their birth (Mehler et al., 1988; Nazzi et al., 1998). Indeed, there is evidence that 6-month-old infants prefer to listen to words of their own language (Jusczyk et al., 1993), and when they can choose whether to listen to a continuous speech stream in their native language or in a foreign language, already 2-days-old newborns prefer to listen to the speech stream in their native language (Moon et al., 1993). Furthermore, newborns’ cries’ melody reflects the melodic contour of their native language (Mampe et al., 2009). This indicates the importance of prenatal experience in speech processing (Partanen et al., 2013). In addition, this early sensitivity to native language speech patterns later results in fine tuned discrimination of the phonetic contrasts of their native language: while between the age of 6 and 12 months infants’ non-native phonetic perception slowly declines, their sensitivity to native-language phonetic contrasts increases (Kuhl et al., 2006).

Importantly, beyond the merely auditory preference for native speech patterns, language perception also plays a role in early social evaluations and preferences. For instance, at the age of 6 months, infants do not only show a preference for their native language, but they also prefer to look at a person, who previously spoke in their language, relative to a person, who spoke in a foreign language (Kinzler et al., 2007). In addition, the same effect was shown also with contrasting native and foreign accents (Kinzler et al., 2007). At 12 months, infants also tend to choose a food that was positively commented by a native speaker, rather than a food that was presented in a context of a similarly positive attitude of a foreign speaker (Shutts et al., 2009). Furthermore, by the age of 5 years, children would rather become friends with a native accented speaker of their language, than with a foreign accented speaker, even if they can understand equally well both speakers (Kinzler et al., 2007).

Language is not the only cue driving social categorization during development. When looking at the emergence of social preferences, it seems that throughout childhood, children categorize others based on their perceptual characteristics such as gender (for a review see Maccoby and Jacklin, 1987), age (French, 1987; Montepare and Zebrowitz, 1998), or ethnic origin (Kowalski and Lo, 2001; Katz, 2003; Kinzler and Spelke, 2011). Nonetheless, when language is pitted against race, children prefer to choose to be friends with a native-accented speaker, even if the person is a member of an other-race group (Kinzler et al., 2009, 2010).

Thus, the question arises: what can be the origins of the privileged status of language that can bias infants’ social preferences from very early on? Linguistic cues, defining ethnic boundaries have a long evolutionary history, as many researchers argue (Cosmides et al., 2003; Henrich and Henrich, 2007; Kinzler et al., 2010; Cohen, 2012; Pietraszewski and Schwartz, 2014a,b). Even though during modern times, both race and language might act as important cues when defining social origins, concerning their evolutionary history, they show fundamental differences. Since in the past long-distance traveling could not exceed the geographic scale of race-defining features, those physical properties that characterize different races were not recurrent features in the environment of our ancestors. Thus, it is quite unlikely that we would have developed a dedicated cognitive system in order to be able to categorize people on the basis of race-related physical properties. In the case of language, however, since linguistic variations could evolve in geographically neighboring areas, even short-distance traveling allowed contact with different speakers (Kelly, 1995; Bowern, 2010). Hence, exposure to different languages and accents was most probably a recurrent feature in the environment of our ancestors, leading to the emergence of a cognitive system that is dedicated to categorize people on the basis of lingusitic cues.

This idea is also supported by empirical research. Pietraszewski and Schwartz (2014a,b) used a memory confusion task, which is a widely accepted method for measuring implicit social categorization (Kurzban et al., 2001; Susskind, 2007). In this task, participants watch faces while they are also listening to simple statements. After a distraction task, they are presented with an array of the previously seen faces and asked to remember which statement was produced by which face. Since the task is very difficult with usually a high error rate, participants’ answers are largely based on their guesses. Importantly, however, most of their errors are due to non-conscious categorization processes, where faces belonging to the same implicit social category are easily mixed, attributing the statement to another face that belongs to the same category. By using this paradign, Pietraszewski and Schwartz (2014a) found that subjects tended to categorize people on the basis of their accent, but not on low-level sound features or familiarity (ease-of-processing).

In addition, another series of experiments with the same paradigm revealed that while categorization by race can be suppressed in case other salient grouping factors are present (resulting a different categorization), accent is still a strong factor that guides implicit categorization (Pietraszewski and Schwartz, 2014b). In these experiments the authors presented again the same characters, who instead of producing neutral statements, gave explicit information about their group membership (i.e., charity groups). Furthermore, as a salient physical property, they were also wearing either a red or a yellow T-shirt. These cues, however, were in conflict both with the information about their accent (in the first experiment) and with the information about their race (in the second experiment). Thus, the critical question was whether categorization based on accent, or based on race would be reduced when these cues are no longer valid predictors of group membership. As the results showed, this was the case for race, but not for accent. Accent, but not race, is thus a dedicated dimension of social categorization, as the authors concluded (Pietraszewski and Schwartz, 2014b).

Tracking social categories and group membership is important in order to be able to predict and guide future social interactions (e.g., Cosmides et al., 2003; Kinzler and Spelke, 2011; Cohen, 2012; Kinzler et al., 2012). Importantly, it seems that even during infancy and early childhood, language-based social categorization can bias prosocial behavior (Kinzler et al., 2012). In their study, Kinzler et al. (2012) showed that both 10-month-old infants and 2,5-year-old children were more likely to engage in collaborative actions with native speakers of their language than with foreign speakers. In the experiments, 10-month-old infants saw videos of two objects being presented by either a native speaker or a foreign speaker, respectively. After the demonstration, the videos froze showing the two speakers holding the two objects, and simultaneously two real exemplars of the videotaped objects were placed in front of the infants. When having the option to choose between these real exemplars, infants reached significantly more often toward the object that was offered by the native speaker. In a second experiment 2,5-year-old children had the opportunity to give an object to one of the two speakers. According to the results, children were more likely to offer the object to the native speaker (Kinzler et al., 2012). Thus, as the authors concluded, already in early development, language is a crucial factor to guide collaboration and prosocial behavior.

Since collaboration is an important characteristic of the human species (Tomasello et al., 2005, 2012; Tomasello and Hamann, 2012), being able to track potential collaborators in complex societies, specifically to represent social categories in order to affiliate oneself with different groups of collaborators, is an evolutionarily adaptive behavior (Cosmides et al., 2003). However, besides finding collaborative partners, tracking language-based social groups might also be essential for social learning. Since sharing the same language indicates belonging to the same social group, most probably it also implies sharing the same cultural background, including conventional tool uses or cultural norms and practices of the group (Csibra and Gergely, 2006; Gergely and Csibra, 2006). Thus, selecting those individuals with whom infants and young children share the same language might also serve the epistemic function of acquiring relevant knowledge of their social group.

Indeed, recent studies provided evidence that both infants and young children can use language as a cue to guide their learning processes. For example, based on the language of a demonstrator they can select which information to imitate or to preferably attend (Buttelmann et al., 2013; Soley and Sebastián-Gallés, 2015). In a study in which 14-month-old infants watched a novel action performed by either a native speaker of their language or a foreign speaker, infants imitated more often the action demonstrated by the native speaker (Buttelmann et al., 2013). Furthermore, infants already at the age of 7-months display a preference for tunes that were introduced by a native speaker of their language, as opposed to tunes that were introduced by a foreign speaker (Soley and Sebastián-Gallés, 2015).

However, if infants’ and young children’s preference for native speakers reflect their motivation to acquire relevant information of their social group, they should first show some attentional selectivity regarding the information they receive from a native speaker of their language, as opposed to the information they receive from a foreign speaker. Thus, we propose that in case infants’ and children’s language-based selective imitation and preference is driven by their motivation to learn information relevant to their social group, this selectivity should be present already at the level of attention, modulating how infants attend information coming from different speakers.

Despite its relevance regarding the potential epistemic function of infants’ and young children’s preference for native speakers, this question has received very little attention in the literature. Thus, the purpose of the current study is to investigate infants’ attentional processes toward the information they receive from native vs. foreign speakers in their environment. Based on the evidence about infants’ selective learning processes from speakers of different languages, we predict that when speakers of different languages provide information, infants will also focus more and explore longer the information coming from a native speaker, as opposed to a foreign speaker, independently from the modality of the information. Furthermore, in case this selectivity of attention is supposed to help the acquisition of relevant information of the social group very early on, we assume that it should be present already in the preverbal period.

To test this prediction, we collected data from eighty 12- and 5-month-old monolingual Italian infants. The infants were first familiarized with two Italian and Slovenian bilingual speakers, one talking to them in Italian and the other talking in Slovenian. After the Familiarization Phase, in the Teaching Phase infants saw clips where the two speakers silently gazed toward two unfamiliar objects, respectively. In the Test Phase, in order to assess infants’ interest and motivation for further exploration toward one or the other object, we presented only the two familiar objects together, while infants’ looking behavior was measured. We predicted that in case they favor the information that is presented by a speaker of their native language, they would attend longer the object presented by this speaker, compared to the object presented by the foreign speaker. In addition, to have an indication of whether they managed to encode the objects, we decided to include movies in the test phase in which we presented each of the previously observed (i.e., familiar) objects together with a novel one. We predicted that in case infants managed to encode the objects, they would prefer to look longer at the novel object, thus show a novelty effect when they see any of the familiar objects together with a novel one.

## Experiment 1

### Materials and Methods

#### Participants

In Experiment 1, we tested 54, 12-month-old infants from Italian-speaking families, (age range: 11 months, 13 days–12 months and 15 days). The age group was chosen because according to studies on object identification, by the age of 12-months infants are definitely able to identify objects based on object property information (Xu, 1999; Xu et al., 1999, 2004; Van de Walle et al., 2000). Six infants were excluded from analysis due to fussiness, and eight were excluded due to insufficient valid trials. Our study was approved by our institutional review board: the Bioethics Committee of the International School for Advanced Studies. All of our experiments followed the guidelines of this committee and all our protocols were approved by the committee. After being informed about the procedure, the parents of all participants provided written consent.

#### Stimuli

Infants were presented with videos of female faces and objects. The videos of the Familiarization Phase consisted two videos of faces of two female speakers, centrally located on the screen, in an approximate life-size. The videos of the Teaching Phase first showed a fixation cross, then one of the faces in the middle and a red occluder on either the right or left side of the screen, which would be later removed to reveal a colorful toy, (approximately 25 cm × 25 cm large). The videos of the Test Phase consisted of two colorful toys, located on the right and left side of the screen.

#### Apparatus and Procedure

Infants sat on a parent’s lap at 80 cm from a 17-inches LCD screen in a soundproof booth. Parents wore opaque eye-glasses to prevent them from seeing the stimuli and influence the infants’ behavior. Stimuli were presented using Psyscope B70 software. Stimuli presentation was controlled from outside the booth by the experimenter. Sound was played from a loudspeaker located behind the screen. Infants were videotaped during the experiment.

A Tobii T-120 Eye-tracker system recorded infants’ gaze position on the screen during the experiment. Before starting the recording, we performed a 5-point calibration. Attractors were presented one after the other on each corner and in the center of the screen. For each attractor, we waited for the position of the gaze to stabilize, before presenting the next one. The difference between the estimated gaze position and the real position of the attractor was used for the calibration. If calibration was not successful it was repeated. No infants were excluded due to failure of calibration. After calibration and during the experiment, infants’ gaze position was recorded at a sampling rate of 60 Hz.

##### Familiarization phase

**FIGURE 1:**
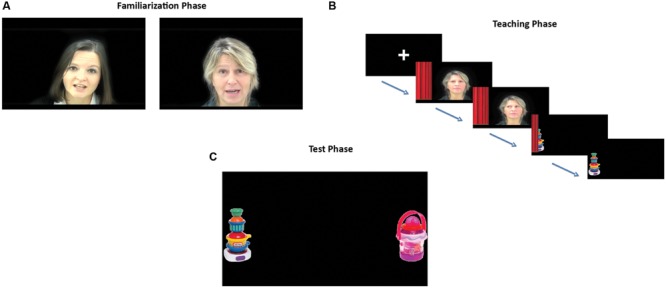
**(A)** Familiarization Phase. Presentation of the two speakers, one of them was speaking in Italian, the other was speaking in Slovenian to the infants. **(B)** Teaching Phase. The two speakers were silently gazing toward an occluder that later revealed an object. **(C)** Test Phase. Presentation of pairs of objects on the two sides of the screen. The combination of the pairs were: Familiar object presented by the Native Speaker vs. Novel object 1; Familiar object presented by the Foreign Speaker vs. Novel object 2, Familiar object presented by the Native Speaker vs. Familiar object presented by the Foreign Speaker.

First, infants went through a Familiarization Phase in which two videos of two female faces were presented, one after the other (**Figure 1A**). Each of these faces was speaking in an infant-directed way while gazing at the infant, for two blocks of 20 s. The blocks of the two speakers alternated and the order of presentation was counterbalanced across subjects. While one of these speakers talked to the infant in her native language (Italian), the other talked to the infant in a foreign language (Slovenian). Both speakers first greeted the infant, then asked her/him about her/his daily activities and told her/him that soon s/he will be watching some nice movies (referring to the experiment) and expressed the hope that the infant will enjoy her/his time in the lab. To control for any possible preference for one face over the other, we recorded Italian–Slovenian bilingual speakers and counterbalanced the language they spoke across infants. The entire Familiarization Phase lasted for 80 s.

##### Teaching phase

After the familiarization, infants were presented with the Teaching Phase consisting of four trials. Each trial started with 5 s of a centrally presented fixation cross. Following this, one of the characters was presented centrally and an occluder was presented either on the left or on the right side of the screen. For 1 s the face was gazing directly at the infant, and after that over a period of 2 s moved the direction of her gaze toward the occluder. After staying there for 2 s the face silently faded away. Once the face disappeared completely, the occluder was removed in a period of 2 s, revealing an object that stayed on screen for 3 s (**Figure 1B**). Since we wanted to eliminate the use of language at this stage, we decided to use the eye-gaze of the two speakers in order to establish a referential relationship between the speakers and the objects. There is evidence showing that young infants and already newborns tend to follow eye-gaze and understand the referential intention of the gaze (e.g., Farroni et al., 2004; Senju et al., 2008; Csibra, 2010; Marno et al., 2015). Thus, by having the speaker gaze toward the object we ensured that infants would make the inference that the speakers are intentionally showing these objects to them.

To avoid possible effects due to side of presentation of the objects, each speaker presented the same object in two different trials, once on each side of the screen. The trials of the Teaching Phase were presented in a random order. The entire Teaching Phase lasted for 60 s.

##### Test phase

The Test Phase consisted of six trials. These trials started with a fixation cross presented centrally for 2.5 s. Afterward, two objects were shown simultaneously, one on the left and one on the right side of the screen, for a period of 10 s. Four objects were presented, out of which two were the objects previously presented during the Teaching Phase, and two were entirely novel objects (**Figure 1C**). Infants saw the test trials in the following combinations: Familiar object presented by the Native Speaker vs. Novel object 1; Familiar object presented by the Foreign Speaker vs. Novel object 2, Familiar object presented by the Native Speaker vs. Familiar object presented by the Foreign Speaker. Each combination was repeated twice in order to balance for the side, thus infants received a total of six test trials that was presented in a random order. The entire Test Phase lasted for 75 s.

#### Data Analysis

We defined three equally sized regions of interest for the analysis of the infant eye gaze, by dividing the screen into a center, a right and a left ROI. Trials of the Teaching Phase had to meet two criteria to be considered valid. First, infants should gaze for at least 2.5 s (out of 5 s) to the center while the character was displayed. Second, infants should gaze for at least 1.5 s (out of 3 s) to the side of the screen where the object was presented. The infants who did not meet these criteria in all the trials of the Teaching Phase were excluded as they likely did not pay sufficient attention to the speaker, to the object, or generally to the movies. For each test trial, we calculated the difference in cumulative looking time to each of the two objects presented. To assess for temporal drift, we obtained for each trial the difference of the timestamp between consecutive samples. Given that the recording rate was at 60 Hz, the time difference between two samples had to be 16.6 ms. We found no significant deviation from this value.

### Results

**FIGURE 2:**
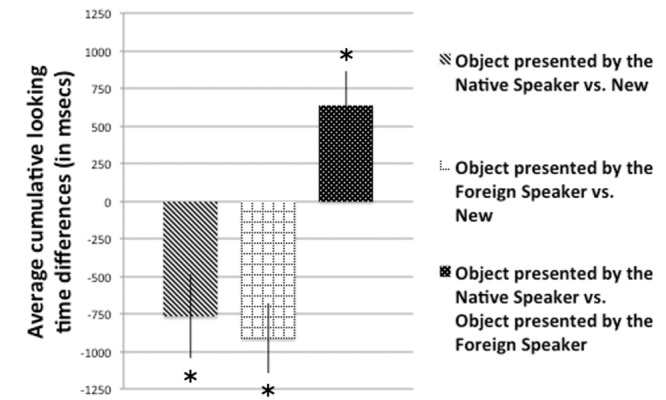
**Average looking time differences in the Test Phase in Experiment 1.** Bars represent the average looking time differences of 40 infants in the three types of test trials. Error bars indicate SEM. Asterisks show significant differences from chance level.

During the Familiarization Phase, infants on average looked at the Native speaker for 18696 ms (*SD* = 322) and for 178040 ms at the Foreign speaker (*SD* = 287). During the Teaching Phase, we analyzed infants’ looking time by comparing average cumulative looking time toward both the object and the speaker in the Native Speaker Condition with the average cumulative looking time toward both the object and the speaker in the Foreign Speaker Condition. This analysis did not produce significant difference, neither in the case of the objects [*t*(39) = 1.501, *p* = 0.141], nor in the case of the speakers [*t*(39) = 1.406, *p* = 0.168]. Thus, independently from the language of the demonstrator, infants looked equally long at both speakers and objects during the Teaching Phase. Next, we calculated the cumulative looking time differences in the Test Phase (**Figure 2**) by extracting the cumulative looking time toward one object minus the other and compared them to chance-level. In both test trials when a novel object was presented, we found a significant novelty effect (in the case of the object presented by the native speaker vs. new object comparison [*t*(39) = -2.690, *p* = 0.010], and in the case of the object presented by the foreign speaker vs. new object [*t*(39) = -3.867, *p* < 0.001]). When the two familiar objects were presented together, infants preferred to look significantly more to the object previously shown by the native speaker [*t*(39) = 2.797, *p* = 0.008]. This difference was also confirmed by a non-parametric binomial test. Out of the 40 infants 28 looked longer at the object that was previously shown by the Native speaker, which was significantly different from chance level (*p* = 0.016). Thus, despite infants spent equally long time to encode the two objects during the Teaching Phase, when they had the opportunity to choose which object to explore more, they preferred to look longer at the object presented by the native speaker of their language. We can conclude that language had a modulatory effect on infants’ object exploratory behavior.

Given that our data suggests that the language spoken by a presenter modulates 12-month-old infants’ object exploration, we wanted to know whether this effect is also present earlier during development. Previous studies showed that already at 6 months of age, infants prefer to look at a person who previously spoke in their native language, as opposed to a person who spoke in a foreign language (Kinzler et al., 2007). Furthermore, 5- and 6-month-old infants showed the same preference toward people who previously spoke to them in a native accent, compared to a person who was speaking in a foreign accent (Kinzler et al., 2007). Thus, if infants’ early preference for native or native accented speakers of their language also serves some epistemic functions, then we might find the same effect also at the age of 5-months. To test this prediction, we ran the same study with 5-month-old infants.

## Experiment 2

### Materials and Methods

The stimuli, apparatus, and protocol we used were identical to Experiment 1.

#### Participants

We tested 61, 5-month-old infants from Italian-speaking families, (age range: 4 months and 15 days to 5 months and 14 days). Ten infants were excluded from analysis due to fussiness, and 11 due to insufficient valid trials.

### Results

**FIGURE 3:**
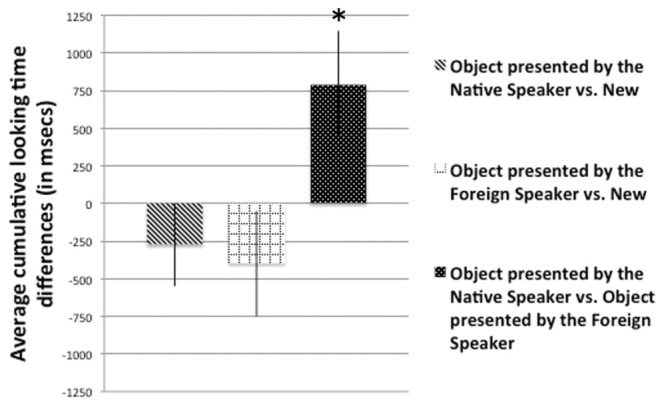
**Average looking time differences in the Test Phase in Experiment 2.** Bars represent the average looking time differences of 40 infants in the three types of test trials. Error bars indicate SEM. Asterisks show significant differences from chance level.

During the Familiarization Phase, infants on average looked at the Native speaker for 19184 ms (*SD* = 208) and for 18976 ms at the Foreign speaker (*SD* = 267). During the Teaching Phase, first we analyzed again infants’ looking time by comparing the average looking time toward both the object and the speaker in the Native Speaker Condition with the average looking time toward both the object and the speaker in the Non-Native Speaker Condition. Just as for the 12-month-olds, this analysis did not yield significant differences, neither in the case of the object [*t*(39) = 0.040, *p* = 0.968], nor in the case of the speaker [*t*(39) = 0.499, *p* = 0.623], indicating that also 5-month-old infants looked equally long at both objects and speakers during the Teaching Phase, independently from the language of the demonstrator. Next, we calculated the total looking time differences in the Test Phase (**Figure 3**) and compared them again to chance level. Even though infants looked numerically longer at the novel object, we did not find a significant novelty effect in any of the two language conditions (in the case of the object presented by the native speaker vs. new object comparison [*t*(39) = -1.002, *p* = 0.323], and in the case of the object presented by the foreign speaker vs. new object [*t*(39) = -1.141, *p* = 0.261]). However, as in Experiment 1, when the two familiar objects were presented together, infants again preferred to look significantly more to the object that was previously shown by the speaker of their native language [*t*(39) = 2.243, *p* = 0.031]. Furthermore, just like in the case of the older age group, this difference was also confirmed by a non-parametric binomial test. Out of the 40 infants 29 looked longer at the object that was previously shown by the Native speaker, which was significantly different from chance level (*p* = 0.0064). Thus, these results show that even 5-month-old infants prefer to attend longer the information they receive from a speaker of their native language, compared to the information they receive from a foreign speaker.

## Comparison of the Two Studies

**Table 1** shows in both age groups infants’ cumulative looking time toward each object in each test condition. In order to directly compare the results of the two age groups in the three test conditions, we conducted a One-Way ANOVA where we included age as a grouping factor. This analysis revealed no difference regarding the looking time differences in the three test conditions [*F*(78) = 1.535, *p* = 0.219 in the comparison of infants’ looking time toward the object that was presented by the Native speaker vs. the Novel object; *F*(78) = 1.482, *p* = 0.227 in the comparison of infants’ looking time toward the object that was presented by the Foreign speaker vs. the Novel object; and *F*(78) = 0.134, *p* = 0.715 in the comparison of infants’ looking time toward the two familiar objects]. Thus, even though in the case of the younger age group we did not find a significant novelty effect when they saw one familiar object together with a novel one, when we compared the two age groups, we did not find a significant difference in any of the test conditions. This might be due to the fact that even though 5-month-olds did not look significantly longer at the novel objects, still numerically they showed a tendency toward having a novelty bias.

**Table 1:** Average cumulative looking time of the two age groups at each object in each test condition.

Age group	Test condition 1		Test condition 2		Test condition 3	
	Object presented by the Native speaker	Novel object	Object presented by the Foreign speaker	Novel object	Object presented by the Native speaker	Object presented by the Foreign speaker
12-month-old group	3348 ms	4109 ms	3149 ms	4065 ms	3947 ms	3310 ms
5-month-old group	3189 ms	3464 ms	3206 ms	3605 ms	3870 ms	3078 ms

*Numbers represent the mean cumulative looking time in milliseconds.*

## Analysis of Infants’ Gaze-Following During the Teaching Phase

There is evidence showing that young infants and even newborns tend to follow eye-gaze and understand the referential intention of the gaze (e.g., Farroni et al., 2004; Senju et al., 2008; Csibra, 2010; Marno et al., 2015). However, in order to be sure that also in our design infants tended to follow the gaze of the speakers during the Teaching Phase, we analyzed their looking behavior in the time window from the moment when the speakers’ eye-gaze reached the direction of the occluder till the moment when the speakers’ faces started to fade away. First, we calculated the percentage of trials in which infants followed the gaze and fixated at the occluder, before it would have revealed the object. This analysis showed that in the 12-month-old group infants followed the eye-gaze in 82% of the trials in the Native speaker condition and in 89% of the trials in the Foreign speaker condition. In the 5-month-old group infants followed the eye-gaze in 82% of the trials in the Native speaker condition and in 73% of the trials in the Foreign speaker condition. Thus, independently of the language of the speaker, infants in both conditions tended to follow the eye-gaze of both speakers. Next, we wanted to see whether the latency of infants’ gaze orientation would differ in the two conditions. Since our data did not follow a normal distribution, instead of calculating the mean value we calculated the median of the latency of gaze-orientation. This analysis revealed in the 12-month-old group a 979 ms median latency of orientation toward the occluder in the Native speaker condition and a 1029 ms median latency of orientation in the Foreign speaker condition. The difference between the two values did not reach a level of significance [*t*(39) = -0.140, *p* = 0.894]. Infants in the 5-month-old group showed a 605 ms median latency of orientation toward the occluder in the Native speaker condition and 456 ms median latency of orientation in the Foreign speaker condition. Again, the difference between the two values did not reach a level of significance [*t*(39) = 1.39, *p* = 0.171].

In sum, infants in both age groups tended to follow the gaze of both speakers to the same extend. Additionally, independently from the language of the speakers, infants oriented equally fast toward the occluder to search for a possible referent of the gaze of the speakers. These results provide additional support to our hypothesis, namely that language only modulates infants’ motivation for further exploration of the received information, and not their general attentiveness.

## Discussion

We proposed that a shared language is a viable cue that can guide infants’ social learning to help to maximize the acquisition of knowledge relevant to the social group of the infant. To test this hypothesis, we designed a preferential looking paradigm in which infants had to attend to objects presented by either speakers of their native language or of a foreign language. We predicted that in case infants are selective information seekers and focus more on the information they receive from a native speaker, then they would prefer to explore more and to look longer at the object that was presented by a native speaker, compared to the object that was presented by a foreign speaker. This prediction was confirmed both in the case of 12- and of 5-month-old infants.

While the older infants always showed a novelty preference when a novel object was presented, when they saw the two familiar objects in a pair, they preferred to look longer at the object that was previously shown by a speaker of their native language. Younger infants, on the other hand, did not show any preference when they saw the familiar objects together with a novel object. However, when the two familiar objects were presented together, similarly to 12-month-old infants, also the 5-month-old subjects preferred to look longer at the object that was presented by the native speaker.

The lack of novelty preference in the group of the younger infants might be because the extent to which they encoded the objects was not sufficient to lead to explicit novelty preference. Previous research on infants’ looking time studies showed that in habituation paradigms, during the course of visual processing infants pass through a period of transition between novelty preference and familiarity preference (Roder et al., 2000; Houston-Price and Nakai, 2004). During this period, however, both the familiar and the novel stimuli attract their attention equally, resulting in no difference in infants’ looking time toward the familiar and the novel stimuli. Thus, this lack of difference, even though it appears as random looking, may in fact be due to two opposing preferences toward the familiar and the novel stimuli (Roder et al., 2000; Houston-Price and Nakai, 2004). Hence, it might be that in our case while the exposure time to the objects was sufficient for the 12-month-old group to lead to a novelty preference, younger infants would have needed more time in order to express an unambiguous novelty preference.

Our results may seem somewhat surprising when compared with the results of Buttelmann et al. (2013) who found that while 14-month-old infants were more likely to imitate the actions of a native speaker, when s/he expressed her/his preference toward an object, infants’ did not rely on this more than on the preference expression of a foreign speaker. How is it possible then that in our case both 12- and 5-month-old infants preferred to look longer at the object that was presented by the native speaker? We believe that this apparent inconsistency of the two studies can be explained by some important differences between the task of Buttelmann et al. (2013) and our task. First, while in the task of Buttelmann et al. (2013) infants were prompted to explicitly choose between two objects by adopting a preference of the model, in our case we only measured their looking time, as an indication of their increased interest toward one object over the other. Thus, while according to the results of Buttelmann et al. (2013) infants did not adopt the preference of the native speaker more often than the preference of the foreign speaker, it remains an open question whether they would still have had an increased interest toward the object that was chosen by the native speaker.

Second, since we applied a within subject design, we could directly contrast the effect of native vs. foreign language, as infants had to choose between the object that was presented by the native speaker and the object that was presented by the foreign speaker. In contrast, in the study of Buttelmann et al. (2013) infants participated only either in the ingroup condition (seeing the attitude expression of the native speaker) or in the outgroup condition (seeing the attitude expression of the foreign speaker). Thus, they never had to choose between the two objects that were presented in the context of the attitude expressions of the two speakers. It thus remains unclear whether during such a forced choice task they would have actually chosen more often the object that was preferred by the native speaker.

One could argue that in our study the longer looking toward the object presented by the native speaker could be due to a novelty effect, because infants might have encoded better the object that was presented by the foreign speaker. However, this alternative explanation would be strikingly at odds with the existing literature. There is abundant evidence that infants from a very early age do not only prefer speakers of their native language (Kinzler et al., 2007), but they also modify their behavior depending on the attitude expressions of speakers of their native language (Shutts et al., 2009). Furthermore, when they have the possibility to accept or to offer objects to different speakers, infants preferably choose the person who previously spoke in their native language, compared to a foreign speaker (Kinzler et al., 2012). Thus, by taking into account infants’ increased preference and affiliative tendencies toward speakers of their native language, one should predict that they would also show a bigger interest, hence a deeper encoding of the object that is presented by a native speaker. Suspecting the opposite, namely that infants would encode better objects that are presented by foreign speakers has no theoretical grounding.

Another alternative explanation of our results could be to assume that the ease of processing the native language with which infants are highly familiar would have led to a general preference toward the native speaker, extended to other information that appeared together with the speaker. Ease of processing or cognitive fluency has been shown to be generally associated with positive attitudes, leading to bigger confidence, trust in the truth of the information and a higher valuation (Alter and Oppenheimer, 2009a,b). Thus, it could be a plausible low-level explanation of our results to assume that infants simply had a preference toward the familiar sound-patterns, compared to the foreign sounds. However, in our paradigm, only during the familiarization phase infants had the opportunity to form an association between the speakers and the languages, leading to a familiarity-based preference toward the native speaker. When we first presented the objects, by the time the object appeared on the screen, the speakers were not present any more. Thus, forming a simple perceptual association between the object and the speaker was not possible. Rather than visually associating the speaker and the object, we believe that infants understood the referential intention of the speaker that was signaled by her eye-gaze (Farroni et al., 2004; Senju et al., 2008; Csibra, 2010; Marno et al., 2015), and as a consequence, they oriented their attention toward the previously gazed-at object. This assumption was also confirmed by the results of the gaze-analysis that showed that infants in both age groups tended to follow the direction of the gaze of both speakers. Finally, contrary to previous studies, where infants’ and children’s object choices took place always in the presence of the speakers (Kinzler et al., 2007, 2012), in our case during the test phase, infants were presented only with the two objects, without the context of the speakers. Thus, even though we cannot completely exclude the possibility that infants’ behavior during the test was influenced by a familiarity effect, we find this possibility unlikely, given that, such a never-ending chain of associations following one familiar stimulus would lead to overgeneralized non-adaptive preferences.

We argue that infants’ longer looking time toward the object previously presented by a native speaker of their language reflects their increased interest in and motivation for further object exploration. More specifically, we propose that the shared language may have supported the inference that the content of the speaker’s communication (i.e., the presented object) might represent information relevant to the social group of the infant, thus worth learning about. This kind of attentional bias and the selectivity to choose between potential sources of information can be a useful heuristic to optimize efficient learning of tool-uses or conventional norms and practices (Csibra and Gergely, 2006; Gergely and Csibra, 2006).

In the literature there is abundant evidence showing that infants are not passive recipients of the transmitted information, but rational learners who can select from who to learn in their social environment (e.g., Koenig et al., 2004; Koenig and Harris, 2005; Mascaro and Sperber, 2009; Tummeltshammer et al., 2014). This selection process can be guided for example by the reliability of both social and non-social informants (Mascaro and Sperber, 2009; Tummeltshammer et al., 2014). While at the age of 16-months infants are able to distinguish between true and false statements and learn from speakers who display relevant intentional cues (Koenig and Harris, 2005), by the age of 3 years they can select informants based on their past history (i.e., whether they provided true information) and learn about novel labels selectively from reliable informants (Koenig et al., 2004). Moreover, their trust can even be reversed if a previously reliable informant later turns out to be unreliable (Scofield and Behrend, 2008).

However, while tracking the reliability can give information about the trustworthiness of the source of knowledge, in order to maximize efficient learning, it is also important to evaluate the content of the transmitted knowledge. And indeed, there is evidence that infants are also sensitive to the perceived consensus between different informants, which can be the basis of tracking information that is universally shared among members of their culture (e.g., Harris and Corriveau, 2011; Chen et al., 2013). For example, young children tend to endorse claims from those persons who previously shared an agreement (Harris and Corriveau, 2011), even if this consensus was achieved between different race members (Chen et al., 2013). Moreover, when children learn from reliable informants, they also encode the information as normatively more appropriate, and they explicitly protest when a third-party acts deviating from the demonstrated norms (Rakoczy, 2008; Rakoczy et al., 2008, 2009; Rakoczy et al., 2008). Thus, from very early on, children show sensitivity to the information that can be seen as part of their cultural norms or a consensus of their environment.

Importantly, though, according to a recent line of research, accepting social partners as relevant sources of information appears to depend also on linguistic cues, even if the information is not verbal. In their study, Kinzler et al. (2010) showed movies about object-function demonstrations to preschool children. When endorsing the object-function, children relied more on the information they received from a native-accented speaker of their language, compared to the information they received from a foreign-accented speaker. Furthermore, this effect remained the same when in a second experiment both demonstrators spoke non-sense speech (Kinzler et al., 2010).

The relationship between sharing the same behavioral norms and linguistic background was also shown in a study by Oláh et al. (2014) who found that 2-year-old children inferences about the language of a demonstrator depend on whether a performed action was in accordance with the conventional norms of the children or not (Oláh et al., 2014). When children saw unusual actions of a demonstrator (e.g., using a fork to comb her hair), later they expected the demonstrator to speak in a foreign language, rather than in the children’s native language. Thus, conventionality and language are both relevant cues to make inferences about a person’s cultural background, influencing children’s selective learning processes.

In accordance with the theoretical claims of this line of research, we propose that in order to be able to become competent members of their social group, infants must acquire the knowledge shared by their society. This requires not only the ability to recognize members of the same cultural background, but also a modulated attention toward the information they receive from these persons. By showing an increased interest and motivation for further exploration, infants and young children can successfully optimize their learning processes and selectively encode the information they receive from a relevant source of their culture. Our study suggests that such an attention modulatory effect is already present in very young infants. The fact that not only 12-month-old, but also 5-month-old infants preferred to explore longer the object they saw presented by a native speaker of their language implies that very young infants’ preference for their native language and for speakers of their language does not only have an importance in social categorization processes, but also in their attention modulation that can potentially lead to successful learning from members of their society. While the present study showed that infants display a modulated attention toward the information they received from a native speaker, further studies should clarify whether this attentional bias would also affect long-term memory processes, leading to a longer-lasting retention of the information young infants receive from members of their cultural society.

## Author Contributions

HM, BG, and JM designed research. HM, JF, and BG performed research. HM, YV, and JF analyzed data. HM, YV, and MN wrote the paper.

## Conflict of Interest Statement

The authors declare that the research was conducted in the absence of any commercial or financial relationships that could be construed as a potential conflict of interest.
